# Ethnopharmacological Validation of Selected *Coleus* and *Plectranthus* spp. For Skin-Related Applications Through In Vitro Biological Assays

**DOI:** 10.3390/plants15131975

**Published:** 2026-06-26

**Authors:** Márcia Santos Filipe, Vera M. S. Isca, Rebeca André, Tânia C. S. P. Pires, Ana Rita Silva, Gabrielle Bangay, Ana María Díaz-Lanza, Ricardo C. Calhelha, Ahmed A. Hussein, Lillian Barros, Patrícia Rijo

**Affiliations:** 1 CBIOS—Research Center for Biosciences and Health Technologies, Lusófona University, 1749-024 Lisbon, Portugal; marcia.filipe@ulusofona.pt (M.S.F.); vera.isca@ulusofona.pt (V.M.S.I.); rebeca.andre@ulusofona.pt (R.A.); gabrielle.bangay@ulusofona.pt (G.B.); 2 Departamento de Ciencias Biomédicas, Facultad de Farmacia, Universidad de Alcalá de Henares, 28805 Alcalá de Henares, Spain; ana.diaz@uah.es; 3 Centro de Quimica Estrutural, Institute of Molecular Sciences, Universidade de Lisboa, 1749-016 Lisboa, Portugal; 4 Centro de Investigação de Montanha (CIMO), Instituto Politécnico de Bragança, 5300-253 Bragança, Portugal; tania.pires@ipb.pt (T.C.S.P.P.); anasilva@ipb.pt (A.R.S.); calhelha@ipb.pt (R.C.C.); lillian@ipb.pt (L.B.); 5 Laboratório Associado para a Sustentabilidade e Tecnologia em Regiões de Montanha (SusTEC), Instituto Politécnico de Bragança, 5300-253 Bragança, Portugal; 6 Departamento de Ciencias Farmacéuticas, Facultad de Farmacia, Centro de Investigación de Enfermedades Tropicales, Instituto de Investigación Biomédica de Salamanca, Universidad de Salamanca, 37007 Salamanca, Spain; 7 Department of Chemistry, Cape Peninsula University of Technology, Bellville 7535, South Africa; mohammedam@cput.ac.za; 8 Instituto de Investigação do Medicamento (iMed.ULisboa), Faculdade de Farmácia, Universidade de Lisboa, 1649-003 Lisbon, Portugal

**Keywords:** biological activity, *Coleus*, HPLC-DAD analyses, *Plectranthus*, skin ailments

## Abstract

*Coleus* Lour. and *Plectranthus* L’Hér. (Lamiaceae) species are recognized as promising sources of bioactive compounds for skin health, supported by their traditional use in skin-related conditions; however, scientific validation of these traditional uses remains limited for several species. In this work, the biological activities of eight *Coleus* and *Plectranthus* species (*C. barbatus*, *C. cylindraceus*, *C. grandidentatus*, *C. hadiensis*, *C. madagascariensis*, *P. ambiguus*, *P. ecklonii*, and *P. fruticosus*) were evaluated to support their ethnomedicinal relevance. Extracts were assessed for general toxicity (*Artemia salina* model), antimicrobial activity (well diffusion and microdilution methods) and antioxidant capacity (DPPH, TBARS and cellular assays). In addition, anti-inflammatory activity (ability to suppress nitric oxide), cytotoxicity in skin cell lines (HaCaT and HFF-1) and inhibition of skin-related enzymes (elastase, tyrosinase and collagenase) were evaluated. Antibacterial activity was predominantly observed against Gram-positive bacteria, with no general toxicity observed. *P. ambiguous* and *P. ecklonii* showed moderate-to-high antioxidant activity, while anti-inflammatory effects were observed for *C. hadiensis* and *C. madagascariensis* and *P. ambiguous*. None of the extracts showed cytotoxicity. Enzyme inhibition assays highlighted *C. cylindraceus* and *P. ecklonii*. HPLC-DAD analysis confirmed the presence of rosmarinic acid. Overall, the findings support the traditional use of *Coleus* and *Plectranthus* species for skin-related conditions and highlight their potential as sources of bioactive compounds for dermatological and cosmeceutical applications.

## 1. Introduction

The skin is the largest organ of the human body and serves as the first protective barrier against environmental aggressors, infections and physical injuries [1]. Besides its protective function, the skin also represents an important route for topical and transdermal drug delivery due to its selective permeability [2]. Consequently, there is growing interest in identifying natural bioactive compounds capable of protecting skin integrity, preventing oxidative stress and inflammation, and supporting the treatment of skin disorders.

Medicinal plants have been widely used in traditional medicine for centuries and remain an important source of therapeutic agents worldwide. Traditional medicinal practices have long relied on topical plant-based preparations for the treatment of skin disorders. For example, the aerial parts of *Plectranthus ecklonii* Benth. are traditionally used by the indigenous communities in Zimbabwe to relieve skin problems and hyperpigmentation [3,4]. In developing countries, herbal preparations continue to serve as an accessible and essential healthcare resource [5,6].

*Plectranthus* L’Hér. (Lamiaceae) genus comprises more than 350 species distributed worldwide [3,7]. The use of *Plectranthus* spp. in traditional medicine dates back many years due to their antifungal, antibacterial and anti-inflammatory properties [3,7,8]. These characteristics highlight the potential of the genus *Plectranthus* as a valuable source of bioactive compounds with pharmaceutical relevance [7]. Approximately 85% of the identified *Plectranthus* species have been described as possessing medicinal properties [3], namely for dermatological applications [3,7,9].

Species of the genus *Plectranthus* are widely recognized as rich sources of essential oils and phenolic compounds with relevant cosmetic and pharmaceutical applications [10]. *Plectranthus* spp. naturally contain an abundance of phenolic compounds and terpenes, namely monoterpenoids, sesquiterpenoids and diterpenoids [3,7]. Previous studies have reported antiproliferative, antitumoral and cytotoxic activity of abietane diterpenes extracted from *Plectranthus* spp. [7]. Furthermore, plant-derived polyphenols have been described as capable of mitigating UV-induced oxidative stress, inflammation and DNA damage associated with skin disorders [4,11,12,13].

Considering the information presented above, several species within the genus *Plectranthus* have been associated with traditional uses for treating wounds, burns, skin infections, and other dermatological conditions. It is important to note, however, that many species formerly classified under *Plectranthus* have been reclassified based on molecular biology studies and are now assigned to the genus *Coleus* Lour [14]. However, despite the increasing number of phytochemical and biological studies concerning these two genera, important gaps remain regarding the experimental validation of their traditional skin-related uses. While some species, such as *Coleus madagascariensis* (Pers.) A. Chev. (synonym *Plectranthus madagascariensis*) and *Plectranthus ecklonii*, have already demonstrated promising antioxidant and antimicrobial activities [5,6], other species remain poorly explored, particularly regarding anti-inflammatory activity, skin enzyme inhibition and cytotoxicity in skin cell models.

In this context, *Coleus hadiensis* (Forssk.) A.J. Paton (synonym *Plectranthus hadiensis*) and *P. ambiguus* (Bolus) Codd were selected due to the limited information available in the literature concerning their biological activities. Additionally, *Coleus barbatus* (Andrews) Benth. ex G. Don (synonym *Plectranthus barbatus*), *Coleus cylindraceus* (Hochst. ex Benth.) A.J. Paton (synonym *Plectranthus cylindraceus*), *Coleus grandidentatus* (Gürke) A.J. Paton (synonym *Plectranthus grandidentatus*), *C. madagascariensis* and *P. ecklonii* were selected based on their reported traditional use for skin ailments, as summarized in Table 1. To the best of our knowledge, this is the first study providing a comparative and integrated evaluation of multiple biological activities across several *Coleus* and *Plectranthus* species, including underexplored taxa, under standardized experimental conditions relevant for dermatological applications. Therefore, the present work aimed to comparatively evaluate the biological potential of eight *Coleus* and *Plectranthus* species through a panel of in vitro assays relevant for skin applications, including antimicrobial, antioxidant, anti-inflammatory, cytotoxicity and skin enzyme inhibition assays, in order to provide scientific support for their ethnomedicinal relevance and identify promising candidates for future dermatological and cosmeceutical applications.

## 2. Results and Discussion

### 2.1. Extraction

In this work, eight *Coleus* and *Plectranthus* species (*Coleus barbatus*—*Cb*, *Coleus cylindraceus*—*Cc*, *Coleus grandidentatus*—*Cg*, *Coleus hadiensis*—*Ch*, *Coleus madagascariensis*—*Cm*, *Plectranthus ambiguus*—*Pa*, *Plectranthus ecklonii*—*Pe*, and *Plectranthus fruticosus*—*Pf*) were selected for a comprehensive evaluation of their biological potential in the context of their traditional use. The eight species of *Coleus* and *Plectranthus* (*Cb*, *Cc*, *Cg*, *Ch*, *Cm, Pa*, *Pe* and *Pf*) were extracted with methanol using ultrasound-assisted equipment. Methanol was selected as the extractor solvent due to its renowned efficacy in extracting phenolic compounds, which can be attributed to greater efficiency of alcohol in disrupting the plant cell walls compared to other solvents [6].

**Figure 1:**
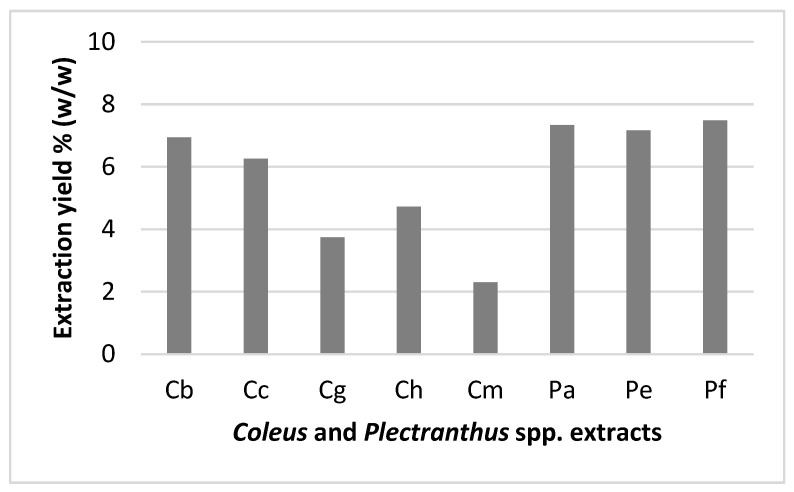
Extraction yield (%, *w*/*w*) of methanolic extracts obtained from eight *Coleus* and *Plectranthus* species using ultrasound-assisted extraction. The eight extracts were tested at 10% (*w*/*w*), where *Cb*, *Coleus barbatus*; *Cc*, *Coleus cylindraceus*; *Cg*, *Coleus grandidentatus*; *Ch*, *Coleus hadiensis*; *Cm*, *Coleus madagascariensis*; *Pa*, *Plectranthus ambiguus*; *Pe*, *Plectranthus ecklonii*; *Pf*, *Plectranthus fruticosus*.

Based on the data presented in Figure 1, the extracts of *Coleus* and *Plectranthus* genera, namely *Pf*, *Pa* and *Pe*, exhibited the most substantial extraction yields, with percentages of 7.5, 7.3 and 7.2% (*w*/*w*), respectively. On the other hand, *Cb*, *Cc* and *Ch* displayed slightly lower extraction yields at 6.9, 6.3 and 4.7% (*w*/*w*), respectively. The extracts *Cg* and *Cm* demonstrated the lowest extraction yields, measuring 3.7 and 2.3% (*w*/*w*), respectively. It should be noted that the long-term storage of the plant material under ambient conditions, despite protection from light, may have led to alterations in its chemical composition, particularly affecting more labile or volatile constituents, and consequently influencing the extraction yields obtained for each plant.

### 2.2. Preliminary Evaluation of General Toxicity on Artemia salina Model

**Figure 2:**
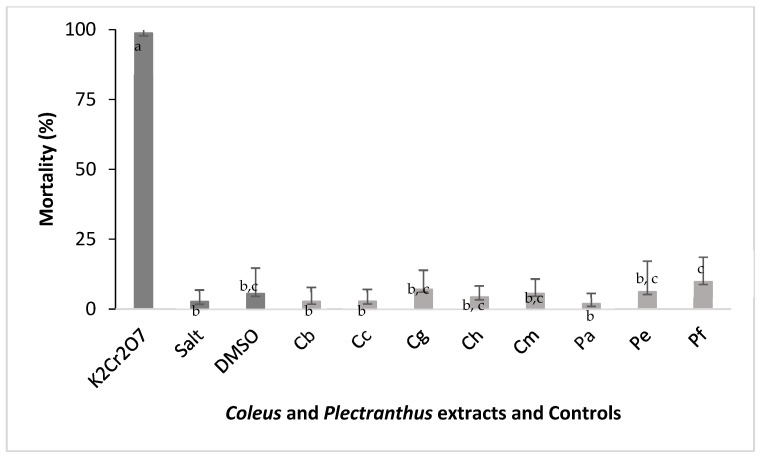
General toxicity assessment of *Coleus* and *Plectranthus* methanolic extracts (0.1 mg/mL) using the *Artemia salina* lethality assay. Results are expressed as percentage mortality after 24 h of exposure and presented as mean ± standard deviation (*n* = 3). Potassium dichromate (K_2_Cr_2_O_7_) was used as positive control, while DMSO (1:100 in salt solution) and saline solution were used as negative and blank controls, respectively. *Cb*, *Coleus barbatus*; *Cc*, *Coleus cylindraceus*; *Cg*, *Coleus grandidentatus*; *Ch*, *Coleus hadiensis*; *Cm*, *Coleus madagascariensis*; *Pa*, *Plectranthus ambiguus*; *Pe*, *Plectranthus ecklonii*; *Pf*, *Plectranthus fruticosus*. Statistical differences were assessed using ANOVA followed by Tukey’s post hoc test. Above columns, different letters next to values indicate significant differences at *p* < 0.05.

The preliminary toxicity assessment of *Coleus* and *Plectranthus* spp. was performed using the in vivo *Artemia salina* model. This assay evaluates the extracts’ potential to induce mortality in the larval stage (nauplii) of microcrustaceans. It provides a quick and initial screening method for the general toxicity of the extracts [27]. According to the data presented in Figure 2, it can be concluded that none of the eight extracts demonstrated toxicity compared to the positive control (potassium dichromate), which resulted in 98.89 ± 2.2% lethality. Since none of the tested extracts induced significant mortality in the larval stage of *A. salina*, it suggests that these extracts may not exhibit acute general toxicity at the tested concentrations. This is a positive finding, as it supports the potential safety of these extracts for further investigation and application in skincare formulations.

### 2.3. Antimicrobial Activity

The antimicrobial activity of the eight extracts was evaluated using the well diffusion method, a commonly applied technique for preliminary screening of plant-derived bioactive compounds [28]. As shown in Table 2, all *Coleus* and *Plectranthus* exhibited activity against Gram-positive bacteria, with inhibition zones ranging from moderate to promising. Specifically, *Pa* and *Cb* extracts demonstrated notable activity against *S. epidermidis*, while *Cg* extract demonstrated the highest activity against both *S. aureus* strains.

In contrast, the activity against Gram-negative bacteria was very limited, with only *Pf* showing a modest inhibition against *E. coli*, and no relevant activity observed against *P. aeruginosa*. Similarly, antifungal activity was weak or absent, with only small inhibition zones detected for *S. cerevisiae* in a few extracts (*Cb*, *Cc*, *Pe*), and no activity against *C. albicans*. These results highlight a clear selectivity of the extracts toward Gram-positive bacteria.

This trend can be explained by structural differences in microbial cell envelopes. Gram-negative bacteria are more resistant than Gram-positive ones due to their outer membrane barrier that acts as an additional permeability barrier, restricting the diffusion of many bioactive compounds [29]. Similarly, fungi’s unique cell wall and membrane sterols can limit the efficacy of bioactive extracts [30]. The evaluation of MIC and MBC values (Table 3) corroborated the well diffusion results, confirming moderate antibacterial activity against Gram-positive strains, particularly for extracts *Cc*, *Cg*, *Pa*, *Pe*, and *Pf*. These extracts showed lower MIC values against *Staphylococcus* spp., supporting their potential relevance. However, high MIC values and a lack of bactericidal effects against Gram-negative bacteria and yeasts further emphasize the restricted antimicrobial spectrum of these extracts when compared to the respective positive controls.

Overall, these findings indicate that while the studied *Coleus* and *Plectranthus* extracts are promising sources of antibacterial agents against Gram-positive bacteria, their limited efficacy against Gram-negative bacteria and fungi should be critically considered when evaluating their potential applications.

### 2.4. Antioxidant Activity

Three different methods were used to evaluate the antioxidant activity of the extracts: DPPH radical scavenging, TBARS and CAA assays. In these assays, quercetin or trolox, two well-known antioxidants [31], were used as control. The antioxidant results are shown in Table 4.

The DPPH method involves measuring antioxidant properties using free radicals to assess the potential of substances to act as free-radical scavengers. The extracts were tested at 0.1 mg/mL and the outcomes were conveyed as the percentage of antioxidant activity at this tested concentration. The *Ce* extract exhibited an exceptionally high antioxidant capacity of 98% when assessed using the DPPH method, surpassing even the positive control quercetin in terms of antioxidant potency. The *Cg* extract exhibited a significant antioxidant activity of 72%, comparable to the positive control, quercetin. Both demonstrated a similar order of magnitude in their antioxidant effectiveness. The *Cm*, *Cc*, *Ch* and *Pa* extracts showed moderate antioxidant activity, ranging between 40 and 50%. In contrast, the *Cb* and *Pf* extracts exhibited low antioxidant activity.

Lipid peroxidation was evaluated using the TBARS assay, which measures malondialdehyde (MDA), a secondary product formed during the oxidation of unsaturated fatty acids. A range of extract concentrations was evaluated, and antioxidant capacity was expressed as EC_50_ values, defined as the concentration at which 50% of lipid oxidation was inhibited. The *Pe* extract exhibited the highest activity, followed by *Cb*, *Cc*, *Cg*, *Ch* and *Pa* extracts. The *Pf* extract displayed the lowest antioxidant activity using this method.

The CAA method was performed to assess the capacity of the extracts to inhibit intracellular oxidation in RAW 264.7 macrophages, using AAPH as a source of peroxyl radicals. Different concentrations of extracts were tested, and the inhibition concentration was determined as the percentage of cells protected from oxidation. The *Pa* and *Pf* extracts exhibited remarkable abilities, protecting 78 and 70% of cells from oxidation, respectively. These results underscore their promising and significant antioxidant activity. The *Cb*, *Cg* and *Pe* extracts displayed moderate activity in this method.

Overall, *Cg* and *Pe* extracts exhibited the most consistent antioxidant performance across the chemical-based assays, particularly DPPH and TBARS, suggesting a strong radical-scavenging and lipid peroxidation inhibitory capacity. The high antioxidant capacity observed for the extracts may be associated with their higher content of phenolic compounds, which are well known for their hydrogen-donating and radical-scavenging properties [32]. This hypothesis is further supported by the phytochemical characterization presented in Section 3.10, where *Cg* and *Pe* extracts exhibited elevated levels of rosmarinic acid, a compound widely recognized for its strong antioxidant activity [33].

These findings align with previous studies on *Coleus* and *Plectranthus* plants, which consistently show that methanol extraction yields abundant quantities of these beneficial compounds [6]. The presence of antioxidant components in the *Coleus* and *Plectranthus* extracts can effectively mitigate oxidative stress and safeguard biomolecules, including proteins, DNA and membrane lipids, from damage caused by free radicals [34].

### 2.5. Anti-Inflammatory Activity

The potential of the extracts as anti-inflammatory agents was evaluated by quantifying their capacity to inhibit the production of the pro-inflammatory mediator nitric oxide (NO) in the LPS-stimulated murine macrophage cell line (RAW 264.7) [35].

The *Ch*, *Cm* and *Pa* extracts demonstrated a moderate capacity to suppress NO production, compared with dexamethasone (positive control) (Table 5). On the other hand, the extracts from *Cc*, *Cg, Pe* and *Pf* did not exhibit any discernible anti-inflammatory activity through this method. Nevertheless, it should be considered that the present method evaluates only nitric oxide production, representing a limited aspect of the inflammatory response. Indeed, the anti-inflammatory potential of different *Coleus* and *Plectranthus* species has been extensively described in traditional medicine and supported by in vitro studies involving other inflammatory pathways, namely the modulation of NF-κB signaling and the regulation of pro-inflammatory cytokines [36,37]. Therefore, the absence of activity in the present assay does not necessarily exclude anti-inflammatory effects mediated through alternative mechanisms.

### 2.6. Cytotoxic Effects in Skin Cell Lines

The cytotoxicity potential of the extracts was assessed in two different skin cell lines: HaCaT and HFF-1 cells (Table 6). Both cell lines were exposed to a range of concentrations of extract to determine their impact in cell viability.

For the HaCaT cell line, the plant extracts *Cc*, *Cg* and *Pe* demonstrated lower IC_50_ values compared to the other extracts, indicating a statistically higher capacity to cause damage to the cells at these concentrations (Table 6). In contrast, *Cm* extract exhibited the highest IC_50_ value, suggesting the lowest cytotoxic effects under the experimental conditions. The observed cell line-dependent effects in HaCaT can be related to differences in phytochemical composition. Extracts such as *Cg* and *Pe*, which showed higher cytotoxic effects in HaCaT cells, have been reported to contain abietane diterpenes, including parviflorone D, a class of compounds associated with cytotoxic activity in various in vitro cell models [38]. On the other hand, *Plectranthus* species are known to be rich in phenolic compounds which are commonly associated with low cytotoxicity in cellular models [39].

In HFF-1 fibroblasts, *Cg* and *Pe* exhibited IC_50_ values of 219.06 ± 21.84 and 384.37 ± 32.92 μg/mL, respectively, while all other extracts showed IC_50_ values above 400 μg/mL, indicating no detectable cytotoxicity at the highest tested concentration. Overall, all extracts displayed low or negligible cytotoxic effects in normal human dermal fibroblasts under the experimental conditions. The primary objective of using plant extracts for skin-related issues is to harness their biological activity without reaching cytotoxic concentrations. The most active extracts in the nitric oxide inhibition assay (*Cm*, *Ch* and *Pa*), evaluated in LPS-stimulated RAW 264.7 macrophages, exhibited IC_50_ values significantly lower than those associated with cytotoxic effects observed in skin cell lines. Although these assays were performed in different cellular models, the results collectively suggest that the anti-inflammatory activity of the extracts occurs at non-cytotoxic concentrations, supporting their potential biological relevance.

### 2.7. In Vitro Skin Enzymes

The elastase assay was performed using SANA as the substrate, by monitoring the release of *p*-nitroaniline at 405 nm, generated through SANA hydrolysis. As observed in Table 7, *Pe* extract, followed by *Cc* extract, demonstrated the highest anti-elastase activity with IC_50_ values of 62.82 ± 1.67 and 84.76 ± 3.56 μg/mL, respectively. The *Pe* showed to be more active than the positive control, kojic acid, with an IC_50_ of 75.55 ± 7.75 μg/mL. The other extracts were unable to inhibit 50% of the enzyme at 100 μg/mL. The high potential of these extracts to inhibit elastase activity was associated with the presence of polyphenols and different diterpenes and pentacyclic triterpenes, which, as previously reported, have already been identified and isolated from *Plectranthus* spp. organic extracts [6,40,41]. Phenolic compounds are known to be competitive inhibitors of elastase due to interactions between their hydroxyl groups and the enzyme active site [42]. Different plants containing different terpenoid compounds, including triterpenes and pentacyclic triterpenoid compounds isolated from *Plectranthus* species, have been described as elastase inhibitors [40]. Our results demonstrated the potential of *Coleus* and *Plectranthus* extracts as an anti-aging agent.

Regarding collagenase inhibition, previous studies have highlighted the role of *Clostridium histolyticum* collagenase (ChC) in collagen degradation, a process associated with skin damage and wrinkle formation [43,44]. In the present study, the extracts *Cg*, *Pe* and *Pf* had the highest anti-collagenase activity with IC_50_ values of 86.62 ± 0.18, 89.36 ± 1.01, and 95.93 ± 5.47 μg/mL, respectively. Similar results have also been obtained for methanolic extracts of *Cg*, *Cm* and *Pe* [40]. Previous studies concerning compound identification and isolation from *Plectranthus* plants are characterized by high levels of phenolic compounds, including rosmarinic, chlorogenic and caffeic acids [6,40,41], which have been reported to exhibit collagenase inhibitory activity [44]. Previous studies reported that catechins, a group of phenolic compounds, can act as a metal chelator, binding Zn^2+^ ion at the active site of ChC enzyme and thereby hindering substrate interaction [40]. Different diterpenes and pentacyclic triterpenes have also been identified in *Plectranthus* spp. organic extracts [6,40]. These groups of compounds have also been characterized with a high capacity to inhibit ChC [40]. This study demonstrated the potential of *Coleus* and *Plectranthus* spp. to prevent skin aging or wrinkles through inhibition of ChC activity.

Mushroom tyrosinase (mTYR) was used to access the capacity of the extracts. As presented in Table 7, no inhibitory activity against tyrosinase was observed for any of the extracts at 100 μg/mL.

### 2.8. HPLC-DAD Analyses

Considering the studied biological activities of *Coleus* and *Plectranthus* spp., the phytochemical profile of the two extracts showing the most consistent antioxidant and skin enzyme inhibitory activities, namely *Cg* and *Pe*, was evaluated by HPLC-DAD to determine the predominant constituents.

**Figure 3:**
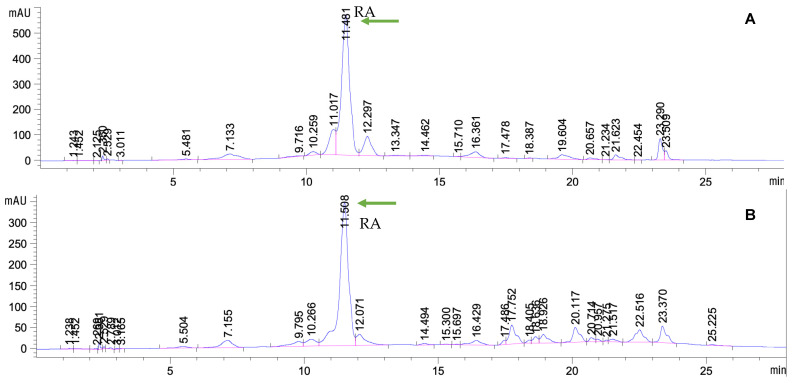
Chromatograms of *Coleus* and *Plectranthus* extracts: (**A**) *Cg*, *Coleus grandidentatus* extract; (**B**) *Pe*, *Plectranthus ecklonii* extract. The wavelength selected was 330 nm. RA—rosmarinic acid was detected in both chromatograms, highlighted with an arrow. The blue trace corresponds to the chromatographic signal and the pink trace to the baseline.

The HPLC-DAD fingerprinting of extracts confirmed the presence of a well-known hydroxycinnamic acid, rosmarinic acid (RA), previously identified in other *Plectranthus* extracts [6]. Since RA was identified as the major compound in both extracts, its content was further quantified using a calibration curve established by analyzing four concentrations of pure RA. The regression analyses demonstrated good linearity, as evidenced by a coefficient of determination (R^2^) of 0.99. The regression equation, retention time (Rt) and R^2^ values obtained for RA in each plant extract are provided in Table 8. The assignment of the RA peak in each chromatogram was achieved based on retention time and UV spectral data. The *Pe* extract demonstrated the presence of 35.3 ± 0.0001 μg/mg of RA (yield of 0.71%), while the *Cg* extract indicated the presence of 26.7 ± 0.0002 μg/mg of RA (yield of 0.53%). The chromatograms of each extract can be consulted in Figure 3. The presence of RA in both extracts may partially explain the biological activities observed in the present study, since *Pe* extract, which exhibited the highest RA content, also demonstrated the strongest antioxidant activity in DPPH and TBARS assays, as well as relevant elastase and collagenase inhibitory activities. Similarly, *Cg* extract showed considerable antioxidant and anti-collagenase activity. Previous studies have described RA as a potent antioxidant and anti-inflammatory phenolic compound with protective effects against oxidative stress and skin aging processes [33]. Nevertheless, the observed bioactivities cannot be exclusively attributed to RA, since other phenolic compounds and terpenoid constituents previously described in *Plectranthus* species may also contribute through synergistic interactions.

Taken together, these findings highlight the relevance of RA as a potential contributor to the observed bioactivities, while also supporting the likelihood of synergistic effects involving other constituents. In this context, it should be acknowledged that the long-term storage of the plant material under ambient conditions may have altered its chemical composition and consequently may have contributed to variations in extraction yields and overall chemical profiles. Therefore, for the most promising extracts of *C. grandidentatus* (*Cg*) and *P. ecklonii* (*Pe*) further studies should include an extensive phytochemical characterization, as comprehensive profiling (e.g., LC-MS) would improve reproducibility and enable a more detailed understanding of their chemical composition.

## 3. Materials and Methods

### 3.1. Plant Materials

*Coleus* and *Plectranthus* species were cultivated at the Institute Superior of Agronomy, within the Parque Botânico da Tapada da Ajuda in Portugal. Plant cuttings were obtained from the Kirstenbosch National Botanical Gardens in South Africa. The selected species were harvested between 2007 and 2010, following which voucher specimens were deposited in the Herbarium “João de Carvalho e Vasconcellos” at the Institute Superior of Agronomy in Portugal. The material collected in 2007 and 2010 was stored in cardboard boxes, protected from light and kept at ambient temperature in a dry environment. These conditions were intended to minimize degradation, particularly photo-induced processes. The plant ethnopharmacological knowledge guided the selection of the plant species: *Coleus barbatus* (Andrews) Benth. ex G. Don (synonym *Plectranthus barbatus*) (*Cb*) voucher 831/2007; *Coleus cylindraceus* (Hochst. ex Benth.) A.J. Paton (synonym *Plectranthus cylindraceus*) (*Cc*) vouchers 107/2008 and 574/2008; *Coleus grandidentatus* (Gürke) A.J. Paton (synonym *Plectranthus grandidentatus*) (*Cg*) voucher 572/2008; *Coleus hadiensis* (Forssk.) A.J. Paton (synonym *Plectranthus hadiensis*) (*Ch*) vouchers 833/2007 and 438/2010; *Coleus madagascariensis* (Pers.) A. Chev. (synonym *Plectranthus madagascariensis*) (*Cm*) vouchers 575/2005, 659/2007, 656/2007, 661/2007, 914/2007 and 658/2007; *Plectranthus ambiguus* (Bolus) Codd (*Pa*) voucher 828/2007; *Plectranthus ecklonii* Benth. (*Pe*) voucher 832/2007; and *Plectranthus fruticosus* L’Hér. (*Pf*) voucher 540/2009. All plant names have been verified according to World Flora Online (https://wfoplantlist.org/).

### 3.2. General Remarks

2,2-diphenyl-1-picrylhydrazyl (DPPH), L-tyrosine, kojic acid, quercetin, tyrosinase from mushroom, N-succinyl-Ala-Ala-Ala-p-nitroanilide (SANA), N-[3-furyl-acryloyl]-Leu-Gly-Pro-Ala (FALGPA), collagenase from *Clostridium histolyticum* type IA, sodium nitrite, dimethyl sulfoxide (DMSO), vancomycin, nystatin, streptomycin, ampicillin, norfloxacin, lipopolysaccharide (LPS), rosmarinic acid ≥ 98% (HPLC) from *Rosemarinus officinalis* L. (R4033), porcine (*Sus scrofa*) brain homogenates, 2,7-dichlorohydrofluorescein (DCFH), malondialdehyde (MDA), thiobarbituric acid (TBA), 2,7-dichlorohydrofluorescein (DCFH) tens and 2,2′-Azobis(2-methylpropionamidine) dihydrochloride (AAPH) was purchased from Sigma-Aldrich (St. Louis, MO, USA). HPLC-grade acetonitrile and methanol and Tris-hydroxymethylaminomethane (Tris) base buffer, potassium dichromate (K_2_Cr_2_O_7_) was bought from VWR International. Trolox was purchased from Fisher Scientific (Waltham, MA, USA). Mueller-Hinton (MHB) and Sabouraud were purchased from Biolab^®^ (Budapest, Hungary). Dulbecco’s Modified Eagle’s medium (DMEM), fetal bovine serum (FBS), Hank’s balanced salt solution (HBSS), L-glutamine, penicillin/streptomycin solutions and trypsin were acquired from Hyclone (Logan, UT, USA). Artemio^®^Salt, Artemio^®^Pur were purchased from JBL GmbH and Co. KG (Neuhofen, Germany). All other chemicals were of analytical grade and purchased from common sources.

All solvents were distilled before use, unless otherwise stated. Dimethyl sulfoxide (DMSO) and HPLC-grade acetonitrile and methanol were used without previous purification. All the tested cell lines were commercially obtained from Leibniz Institute DSMZ—German Collection of Microorganisms and Cell Cultures GmbH (Braunschweig, Germany).

### 3.3. Extraction Procedure of Coleus and Plectranthus spp.

The hole plant (5 g), including leaves, stems and roots of each plant were air-dried and then were fragmented into small pieces for extraction. The extraction process was conducted once using methanol (50 mL, 10% *w*/*v*), in an ultrasonic (US) bath (Sonorex Super RK 510 H; Bandelin, Berlin, Germany) for 15 min (five turns in US) at 25 °C. Filtration was subsequently carried out, followed by evaporation of the solvent under low pressure at 40 °C. The extraction yield (% *w*/*w*) of each extract was calculated, according to the equation:

$$\mathrm{yield}\;(\%)\;=\;\frac{extract\;mass\;\left(g\right)}{plant\;mass\;(g)}\times 100$$

### 3.4. Assessment of General Toxicity Using Artemia Salina Model

The preliminary toxicity of the eight extracts was investigated using *Artemia salina* L. assay [27]. Brine shrimp cysts were hatched in a hatching vessel with an artificial saline solution (35 g/L). For 48 h, at a stable temperature between 22 and 29 °C, cysts hatched in the saline solution under sustained aeration and constant light. All the extracts were tested at 0.1 mg/mL in saline solution. The negative control used was DMSO at 0.1 mg/mL in artificial saline solution and potassium dichromate (K_2_Cr_2_O_7_) (1 mg/mL) in artificial saline solution was used as the positive control. Each well of a 24-well plate was filled with 10–15 nauplii (shrimp larvae) in saline water (900 µL). Then, each sample was added (100 µL), followed by incubation at 25 °C under illumination for 24 h. After the 24 h incubation, the number of dead nauplii in each well was determined using a microscope (12×). Potassium dichromate solution was added to exterminate the remaining nauplii and then the mortality in each well was quantified under a microscope. The mortality rate was calculated using the following equation.

$$Mortality\;Rate\left(\%\right)=\frac{\left(Total\;A.\;salina-Living\;A.\;salina\right)}{\left(Total\;A.\;salina\right)}\times 100$$

All tests were performed in triplicates, with internal quadruplicates. Standard deviations (SD) were computed, and the outcomes were presented as means of replicates, along with the corresponding SD. Artificial salt solution was used as the control sample (blank).

### 3.5. Evaluatiion of Antimicrobial Activity

#### 3.5.1. Well Diffusion Method

The eight extracts were screened for antimicrobial potential through the well diffusion method, as reported by CLSI [45]. The extracts were subjected to testing against Gram-positive bacteria (*Staphylococcus epidermidis* ATCC 12228, *Staphylococcus aureus* ATCC 25923 and ATCC 6538 and *Propionibacterium acnes* ATCC 11827), Gram-negative bacteria (*Escherichia coli* ATCC 25922 and *Pseudomonas aeruginosa* ATCC 27853), and yeast (*Candida albicans* ATCC 10231 and *Saccharomyces cerevisiae* ATCC 2601) strains. The extract samples were prepared at a concentration of 10 mg/mL in DMSO. As positive controls, reference antibiotics were tested at a concentration of 1 mg/mL, namely nystatin for yeast strains, norfloxacin and streptomycin for Gram-negative bacteria, and vancomycin and ampicillin for Gram-positive bacteria. DMSO served as the negative control.

In this assay, agar plates (Mueller–Hinton or Sabouraud for bacteria and yeast strains, respectively) were inoculated with a standardized suspension of the test microorganisms adjusted to a 0.5 McFarland standard. Wells approximately 5 mm diameter were subsequently made in the solid medium to allow the addition of samples and controls (50 μL each). The plates were then incubated for 24 h at 37 °C. All the experiments were conducted in triplicate.

#### 3.5.2. Determination of Minimum Inhibitory Concentration (MIC) and Minimum Bactericidal Concentration (MBC)

The antibacterial potential of all the extracts was also evaluated by the microdilution method [46] and the MIC was assessed in a 96-well microplate, with serial dilutions of the tested samples and 10 µL of bacterial suspension (0.5 McFarland units). The negative control used was DMSO and the positive controls were the reference antibiotics described. Microplates were incubated at 37 °C for 24 h. All the assays were done in triplicate.

To determine the MBC, a swab sample from the selected wells was taken from the MIC determination microplate and seeded into an agar plate. The plates were incubated at 37 °C for 24 h and the minimum concentration at which no visible growth observed was noted as the MBC. All the assays were performed in triplicate.

### 3.6. Assessment of Antioxidant Activity

#### 3.6.1. DPPH Free-Radical Scavenging

The antioxidant capacity of the extracts was assessed using the 2,2-diphenyl-1-picrylhydrazyl (DPPH) radical scavenging assay, as previously described by Ntungwe et al. [46]. The extracts were tested at 0.1 mg/mL and quercetin was used as positive control. All assays were performed in triplicate, and results are expressed as mean ± SD.

#### 3.6.2. Thiobarbituric Acid Reactive Substances (TBARS)

The antioxidant capacity of the extracts was also evaluated through the TBARS method. The extracts were diluted within the range of 250 to 0.24 mg/mL in water. To assess the inhibition of lipid peroxidation in porcine (*Sus scrofa*) brain homogenates, TBARS formation was quantified by measuring the absorbance of the malondialdehyde (MDA)–thiobarbituric acid (TBA) complex at 532 nm. The inhibition ratio in % was considered using the equation [(A − B)/A] × 100%, where A represents the absorbance of the control solution and B represents the absorbance of the sample solution. The results are expressed as EC_50_ values (mg/mL), corresponding to the concentration that provides 50% of the antioxidant activity [47]. All assays were performed in triplicate, and results are expressed as mean ± SD.

#### 3.6.3. Cellular Antioxidant Activity (CAA)

The extract samples at 8 mg/mL were dissolved in a combination of DMSO:water (50:50, *v*/*v*). A working solution of 2,7-dichlorohydrofluorescein (DCFH) in ethanol and HBSS was prepared at 50 µM. The tested samples ranged from 2 to 0.125 mg/mL. This assay followed the protocol defined by de la Fuente et al. [48].

RAW 264.7 murine macrophages cell line (ECACC 91062702 obtained from Leibniz Institute DSMZ, Germany) were cultured in DMEM culture medium supplemented with 10% (*v*/*v*) heat-inactivated fetal bovine serum, 1% (*v*/*v*) penicillin/streptomycin and L-glutamine (2 mM). The cells were maintained at 37 °C in a humidified atmosphere of 5% CO_2_ and used at 70–80% confluence. Cells were separated and adjusted to the initial density of 7 × 10^4^ cells/mL using an automated cell counter (Scepter TM Handheld, Millipore, Billerica, MA, USA). Aliquots of 300 µL of cell solution were seeded into a black 96-well plate with clear bottom (SPL Lifesciences) and incubated for 48 h. After the incubation, the medium was aspirated and the cells were washed twice with 100 µL of HBSS. Next, the cells were subjected to the different extract concentration (200 µL; 2–0.125 mg/mL) for 1 h. Subsequently, the cells underwent two consecutive rinses with 100 µL of HBSS each, and 100 µL solution of 2,2-azobis(2-methylpropionamide) dihydrochloride (AAPH) at 600 µM was added.

Fluorescence was recorded every 5 min over a period of 1 h (SynergyH1, BioTek Instruments, Winooski, VT, USA) with excitation and emission at 485 nm at 538 nm, respectively. As positive control, quercetin was used while dichlorohydroflurescein and DMEM culture medium were used as negative control. Results are expressed as percentage inhibition at the highest tested concentration [48,49]. All assays were carried out in triplicate, and data are presented as mean ± SD.

### 3.7. Determination of Anti-Inflammatory Activity

The anti-inflammatory effect of the extracts was evaluated by assessing their ability to suppress nitric oxide (NO) production induced by lipopolysaccharide (LPS) in RAW 264.7 murine macrophages (Griess Reagent System Kit, Promega, Madison, WI, USA) as previously described [49]. The extracts were prepared at different concentrations (0.125–8 mg/mL) in DMSO:water. A positive control of dexamethasone (50 µM) (Sigma-Aldrich, USA) was employed, while cells without LPS were used as negative control (Sigma-Aldrich, USA). The inhibitory activity was expressed as IC_50_ (µg/mL), corresponding to the concentration needed to reduce NO production by 50% ([35]. The assay was performed in three independent replicates, and results are expressed as mean ± SD.

### 3.8. Evaluation of Cytotoxic Effects in Skin Cell Lines

Cytotoxic effect of the extracts in study was assessed using the sulforhodamine B (SRB) assays, as previously described by Guimarães et al. [49]. The HaCaT immortalized human keratinocyte cell line (ACC 771) was obtained from the Leibniz Institute DSMZ—German Collection of Microorganisms and Cell Cultures GmbH (Braunschweig, Germany), while the HFF-1 human foreskin fibroblast cell line (ATCC^®^ SCRC-1041™) was obtained from the American Type Culture Collection (ATCC, Manassas, VA, USA). Cells (90 μL; 2.0 × 104 cells/well) were incubated overnight to adhere. Then cells were exposed to 10 μL of different extract concentrations (400, 200, 100 and 50 µg/mL) incubated for 48 h. A constant DMSO concentration of 1% was maintained across all samples. As negative control we used media with 1% DMSO which was considered 100% viability, while Triton X served as the positive control (0% viability). After treatment, cells were fixed with cold 10% trichloroacetic acid solution for 60 min at 4 °C. Bound SRB absorbance was measured at 565 nm using a SPECTROstar Nano Multi-Detection Microplate Reader (BMG Labtech, Ortenberg, Germany). All assays were carried out in triplicate, results were confirmed microscopically and analyzed using Graph Pad Prism Software (Graph Pad Prism version 10.0.2 for Windows, San Diego, CA, USA). Data are expressed as mean ± SEM of three independent experiments. IC_50_ values were calculated as the concentration required to achieve a 50% reduction in cell viability.

### 3.9. In Vitro Skin Enzymes Inhibition Assays

#### 3.9.1. Elastase Inhibition Activity

The elastase enzymatic was evaluated using an optimized spectrophotometric assay previously described [40], with some modifications. To perform this assay, 10 µL of the sample (positive control, negative control or extracts), 150 µL of Tris-HCl buffer (50 mM, pH 8.0) and 20 µL of elastase (3 U/mL) were mixed in a 96-well plate. The plates were incubated at 25 °C for 10 min, followed by the addition of the substrate *N*-Succinyl-Ala-Ala-Ala-*p*-nitroanilide (SANA) (1 mM) to start the reaction. Epigallocatechin gallate (EGCG) (100 µg/mL) was used as positive control.

A control assay representing 100% activity was performed by replacing the sample with an equal buffer volume.

Absorbance at 405 nm was recorded at 30 s intervals over a 3 min period, reflecting p-nitroaniline formation from SANA hydrolysis, in a microplate reader (Biotek Synergy HT Microplate Reader, BioTek Instruments, Winooski, VT, USA). The percentage of elastase inhibition (I) was calculated based on the initial reaction velocities, where V_sample_ and V_control_ correspond to the initial rates in the presence and absence of the sample, respectively, following the equation:

$$I\;(\%)\;=\;\frac{\left(Vcontrol-Vsample\right)}{Vcontrol}\times 100$$

All assays were conducted in two independent experiments, each performed in triplicate. Microsoft^®^ Excel 2016 was used for data, and the results are expressed as IC_50_ ± SD. Additionally, software developed by Microsoft^®^ was used to perform statistical analysis using one-way analysis of variance (ANOVA) followed by Tukey’s post hoc test, with *p* < 0.05.

#### 3.9.2. Collagenase Inhibition Activity

Collagenase enzymatic activity was measured using the method described by Andrade et al. [4]. As positive control we used EGCG (100 μM), a strong inhibitor of collagen degradation, while as negative control we used the DMSO 1% (*v*/*v*) solvent samples. The assay was performed in triplicate. Data and statistical analysis using one-way analysis of variance (ANOVA) followed by Tukey’s post hoc test, with *p* < 0.05, was performed using Microsoft^®^ Excel. The results are expressed as IC_50_ ± SD.

#### 3.9.3. Tyrosinase Inhibition Activity

Tyrosinase activity was evaluated by monitoring L-Dopa oxidation using mushroom tyrosinase (mTYR) following the method previously described by Silvia et al. [49] with some modifications. Assays were conducted in 96-well plates in triplicate. Each reaction mixture contained sample solution (7 μL; 100–12.5 µg/mL in 1% DMSO), phosphate buffer (0.1 M, pH 6.8; 119 μL), mTYR (7 μL; 142 U/mL), and L-DOPA (67 μL; 5 mM). Control reactions were prepared by replacing the sample with solvent, while background controls contained buffer and substrate only. 4-n-butylresorcinol (0–20 μM) was used as positive control. The formation of dopachrome was monitored at 475 nm at 37 °C using a microplate reader (Biotek FLX800, BioTek Instruments, Winooski, VT, USA). Enzyme inhibition was determined from the slope of the kinetic curves, using the best-fit linear region, and normalized against control and background signals. Data is presented as mean ± SD.

All assays included appropriate negative and positive controls, and activity was evaluated comparatively, using the negative control as baseline and the positive control as reference for maximal effect.

### 3.10. HPLC-DAD Analysis

The comparison of composition of the plant extracts was assessed through high-performance liquid chromatography (HPLC). The HPLC (Agilent Technologies 1260 Infinity Series LC system, Agilent Technologies, Santa Clara, CA, USA) was coupled with a diode array detection (DAD) [6]. The instrument was equipped with a reverse phase LiChrospher^®^ 100 RP-18 5 μm (4 × 250 mm) column from Merck Millipore, Darmstadt, Germany. The elution process was carried out as described in [6], at 1 mL/min, 29 °C with detection occurring at 254, 270 and 330 nm. Furthermore, the primary constituent of the extracts was identified by comparison with a pure rosmarinic acid standard, considering the retention time and the UV spectra. The pure standard (1 mg/mL) was dissolved in methanol and analyzed in the same analytical conditions as the extracts. All samples were analyzed in triplicate.

## 4. Conclusions

The present study provided a comparative biological evaluation of eight *Coleus* and *Plectranthus* species traditionally associated with skin-related applications. Overall, the obtained results demonstrated that several of the tested extracts possess relevant biological activities associated with dermatological potential, including antioxidant, antimicrobial, anti-inflammatory and skin-related enzyme inhibitory activities.

Among the evaluated species, *Plectranthus ecklonii* demonstrated particularly promising antioxidant and anti-elastase activities, while *Coleus hadiensis*, *Coleus madagascariensis* and *Plectranthus ambiguus* exhibited relevant anti-inflammatory effects. Additionally, *Coleus grandidentatus* and *P. ecklonii* showed noteworthy antibacterial activity and collagenase inhibition. Importantly, the tested extracts did not demonstrate significant toxicity in the *Artemia salina* model or marked cytotoxicity in skin cell lines at biologically relevant concentrations, supporting their potential suitability for future topical applications.

The observed biological activities were generally consistent with the traditional medicinal uses summarized in Table 1, particularly regarding wound healing, skin infections, inflammation and skin protection. Furthermore, the identification of rosmarinic acid in the most active extracts suggests that phenolic compounds may partially explain the antioxidant and skin enzyme inhibitory activities observed in this study.

Despite these promising findings, the present work represents a preliminary in vitro screening study. Therefore, further investigations are required to confirm the dermatological applicability of these extracts, including phytochemical isolation studies, formulation development, skin permeation evaluation, irritation and sensitization assays, as well as ex vivo and in vivo studies. Nevertheless, this work contributes to the scientific validation of the ethnopharmacological relevance of *Plectranthus* species and highlights their potential as promising sources of bioactive compounds for future dermatological and cosmeceutical applications.

## Figures and Tables

**Table 1:** Ethnopharmacological uses, plant parts employed and traditional dermatological applications reported for the selected *Coleus* and *Plectranthus* species evaluated in this study.

Species	Part of the Plant Used	Ethnobotanical Skin Uses	References
*Coleus barbatus* (Andrews) Benth. ex G. Don	Leaves	Edema, wounds, ringworms, mycosis	[15,16]
	Crushed leaves with water	Wounds, edematous area	[16]
	Leaves	Stiffness and muscular cramp	[16]
	N.S.	Eczema	[17]
	Essential oils	Anti-allergic activity	[18]
*Coleus cylindraceus* (Hochst. ex Benth.) A.J. Paton	Leaves	Antiseptic and deodorant dressing for wounds	[19]
	N.S.	Skin diseases (ulcers)	[7]
	Leaves	Disinfectant, fragrant and deodorant	[20]
*Coleus grandidentatus* (Gürke) A.J. Paton	N.S.	Skin treatment	[4]
*Coleus madagascariensis* (Pers.) A. Chev.	Leaves	Scabies and small wounds	[21,22,23,24]
*Plectranthus ecklonii* Benth.	Aerial part of the plant	Skin issues and hyperpigmentation	[3,4]
	N.S.	Skin infection	[25]
*Plectranthus fruticosus* L’Hér.	Leaves	Healing properties, burns	[26]

N.S., not specified.

**Table 2:** Antimicrobial activity using the well diffusion method to evaluate *Coleus* and *Plectranthus* spp. methanol extracts. The extracts were tested at 10 mg/mL, while the positive controls were tested at 1 mg/mL. The results are expressed in inhibition halos (mm).

	*S. aureus* ATCC 25923	*S. aureus* ATCC 6538	*S. epidermidis* ATCC 12228	*E. coli* ATCC 25922	*P. aeruginosa* ATCC 27853	*S. cerevisiae* ATCC 2601	*C. albicans* ATCC 10231
*Cb*	10	11	18	na	na	7	na
*Cc*	11	12	11	na	na	7	na
*Cg*	15	15	11	na	na	na	na
*Ch*	9	13	11	na	na	na	na
*Cm*	8	9	11	na	na	na	na
*Pa*	11	13	16	na	na	na	na
*Pe*	11	12	13	na	na	8	na
*Pf*	10	10	14	11	na	na	na
Positive control	26	25	27	17	33	31	31
	VAN	VAN	VAN	NOR	NOR	NYS	NYS

*S. aureus*, *Staphylococcus aureus*; *S. epidermidis*, *Staphylococcus epidermidis*; *P. acnes*, *Propionibacterium acnes*; *C. albicans*, *Candida albicans*; *S. cerevisiae*, *Saccharomyces cerevisiae*; *E. coli*, *Escherichia coli*; *P. aeruginosa*, *Pseudomonas aeruginosa*; *Cb*, *Coleus barbatus*; *Cc*, *Coleus cylindraceus*; *Cg*, *Coleus grandidentatus*; *Ch*, *Coleus hadiensis*; *Cm*, *Coleus madagascariensis*; *Pa*, *P. ambiguus*; *Pe*, *P. ecklonii*; *Pf*, *P. fruticosus*; na, not active. Negative control: DMSO. Positive controls: VAN, vancomycin (Gram +); NYS, nystatin (yeast); NOR, norfloxacin (Gram −).

**Table 3:** Determination of Minimum Inhibitory Concentrations (MICs) and Minimum Bactericidal Concentrations (MBCs) of *Coleus* and *Plectranthus* spp. extracts in µg/mL.

	***S. aureus*** **ATCC 25923**		***S. aureus*** **ATCC 6538**		***S. epidermidis*** **ATCC 12228**		***P. acnes*** **ATCC 11827**		***E. coli*** **ATCC 25922**		***P. aeruginosa*** **ATCC 27853**		***S. cerevisiae*** **ATCC 2601**		***C. albicans*** **ATCC 10231**	
	**MIC**	**MBC**	**MIC**	**MBC**	**MIC**	**MBC**	**MIC**	**MBC**	**MIC**	**MBC**	**MIC**	**MBC**	**MIC**	**MBC**	**MIC**	**MBC**
*Cb*	78.1	625	78.1	<625	78.1	<1250	5000	>10,000	625	2500	5000	>10,000	312.5	2500	625	2500
*Cc*	78.1	312.5	39.1	625	39.1	<625	2500	>10,000	625	1250	5000	>10,000	625	2500	625	2500
*Cg*	78.1	625	19.5	625	19.5	625	2500	>10,000	312.5	1250	5000	>10,000	312.5	2500	31.2	2500
*Ch*	78.1	625	78.1	1250	78.1	625	10000	>10,000	625	5000	5000	>10,000	312.5	5000	625	2500
*Cm*	156.2	<1250	78.1	<625	78.1	<1250	n.t.	n.t.	625	2500	n.t.	n.t.	625	2500	625	2500
*Pa*	78.1	<1250	19.5	<625	19.5	625	5000	>10,000	625	2500	1250	>10,000	312.5	2500	625	2500
*Pe*	78.1	625	19.5	<625	19.5	<156.2	5000	>10,000	625	2500	2500	>10,000	312.5	2500	625	2500
*Pf*	156.2	1250	39.1	625	78.1	625	2500	>10,000	625	2500	10,000	>10,000	625	5000	625	2500
Positive control	1.95	500	>0.49	<500	1.95	62.5	0.07	5	0.49	15.6	0.06	0.06	1.95	62.5	0.98	500
	VAN		VAN		VAN		AMP		NOR		STREP		NYS		NYS	

*S. aureus*, *Staphylococcus aureus*; *S. epidermidis*, *Staphylococcus epidermidis*; *P. acnes*, *Propionibacterium acnes*; *C. albicans*, *Candida albicans*; *S. cerevisiae*, *Saccharomyces cerevisiae*; *E. coli*, *Escherichia coli*; *P. aeruginosa*, *Pseudomonas aeruginosa*; *Cb*, *C. barbatus*; *Cc*, *C. cylindraceus*; *Cg*, *C. grandidentatus*; *Ch*, *C. hadiensis*; *Cm*, *C. madagascariensis*; *Pa*, *Plectranthus ambiguus*; *Pe*, *Plectranthus ecklonii*; *Pf*, *Plectranthus fruticosus*; n.t., not tested. Negative control: DMSO. Positive controls: VAN, vancomycin and AMP, ampicillin (Gram +); NYS, nystatin (yeast); NOR, norfloxacin and STREP, streptomycin (Gram −).

**Table 4:** Antioxidant activity of *Coleus* and *Plectranthus* spp. extracts using three different methodologies, DPPH, TBARS and CAA. Positive controls: quercetin (DPPH and CAA); trolox (TBARS).

Samples	DPPH (% Antioxidant Activity at 0.1 mg/mL)	TBARS (EC_50_, mg/mL)	CAA (% Inhibition of Oxidation at 2 mg/mL)
*Cb*	32.11 ± 0.02 ^b^	0.043 ± 0.003 ^b^	59 ± 9.79 ^a,b^
*Cc*	51.23 ± 0.08 ^c^	0.043 ± 0.001 ^b^	43 ± 11.87 ^b^
*Cg*	72.34 ± 0.01 ^f^	0.051 ± 0.001 ^e^	56 ± 9.66 ^a,b^
*Ch*	43.71 ± 0.00 ^g^	0.093 ± 0.002 ^f^	49 ± 4.89 ^b,c^
*Cm*	52.03 ± 0.06 ^h^	n.t.	>2000
*Pa*	40.10 ± 0.04 ^a^	0.086 ± 0.004 ^a^	78 ± 6.7 ^a^
*Pe*	98.15 ± 0.00 ^d^	0.038 ± 0.003 ^b^	58 ± 12.85 ^a,b^
*Pf*	23.55 ± 0.04 ^e^	0.30 ± 0.01 ^c^	70 ± 8.55 ^a,c^
Positive control	79.75 ± 0.04	0.0058 ± 0.0006	95.3 ± 4.6

where *Cb*, *Coleus barbatus*; *Cc*, *Coleus cylindraceus*; *Cg*, *Coleus grandidentatus*; *Ch*, *Coleus hadiensis*; *Cm*, *Coleus madagascariensis*; *Pa*, *Plectranthus ambiguus*; *Pe*, *Plectranthus ecklonii*; *Pf*, *Plectranthus fruticosus*. n.t.—not tested. Positive controls: quercetin (DPPH and CAA); trolox (TBARS). Statistical differences were assessed using ANOVA followed by Tukey’s post hoc test. In columns, different letters next to values indicate significant differences at *p* < 0.05.

**Table 5:** Anti-inflammatory activity of *Coleus* and *Plectranthus* extracts using RAW 264.7 macrophages. IC_50_ results are present in μg/mL and expressed as mean ± standard deviation.

Samples	NO Production Inhibition (IC_50_, µg/mL)
*Cb*	228.52 ± 4.98 ^b^
*Cc*	>400
*Cg*	>400
*Ch*	57.10 ± 1.44 ^c^
*Cm*	54.45 ± 3.63 ^c^
*Pa*	71.89 ± 5.11 ^a^
*Pe*	>400
*Pf*	>400
Dexamethasone	6.3 ± 0.4

where *Cb*, *Coleus barbatus*; *Cc*, *Coleus cylindraceus*; *Cg*, *Coleus grandidentatus*; *Ch*, *Coleus hadiensis*; *Cm*, *Coleus madagascariensis*; *Pa*, *Plectranthus ambiguus*; *Pe*, *Plectranthus ecklonii*; *Pf*, *Plectranthus fruticosus.* Positive control: dexamethasone. Statistical differences were assessed using ANOVA followed by Tukey’s post hoc test. Different letters next to values indicate significant differences at *p* < 0.05.

**Table 6:** Cytotoxic effects of the *Coleus* and *Plectranthus* spp. against two skin cell lines. IC_50_ results expressed in μg/mL.

	Cytotoxic Activity (IC_50_ μg/mL)	
Samples	HaCaT	HFF-1
*Cb*	120.42 ± 3.36 ^b^	>400
*Cc*	81.92 ± 4.51 ^a^	>400
*Cg*	56.80 ± 3.48 ^a^	219.06 ± 21.84 ^b^
*Ch*	197.74 ± 13.14 ^d^	>400
*Cm*	339.61 ± 14.60 ^e^	>400
*Pa*	153.03 ± 7.29 ^c^	>400
*Pe*	73.45 ± 4.34 ^a^	384.37 ± 32.92 ^a^
*Pf*	180.85 ± 11.43 ^d^	>400

Statistical differences were assessed using ANOVA followed by Tukey’s post hoc test. In columns, different letters next to values indicate significant differences at *p* < 0.05. *Cb*, *Coleus barbatus*; *Cc*, *Coleus cylindraceus*; *Cg*, *Coleus grandidentatus*; *Ch*, *Coleus hadiensis*; *Cm*, *Coleus madagascariensis*; *Pa*, *Plectranthus ambiguus*; *Pe*, *Plectranthus ecklonii*; *Pf*, *Plectranthus fruticosus*. The media with 1% DMSO was used as negative control (100% viability), while Triton X served as the positive control (0% viability).

**Table 7:** Inhibition activity of three skin enzymes: elastase, collagenase and tyrosinase.

	IC_50_ Value (μg/mL)		
Sample	Elastase Inhibition Activity	Collagenase Inhibition Activity	Tyrosinase Inhibition Activity
*Cb*	>100	>100	>100
*Cc*	84.76 ± 3.56 ^a^	>100	>100
*Cg*	>100	89.36 ± 1.01 ^a,b^	>100
*Ch*	>100	>100	>100
*Cm*	>100	>100	>100
*Pa*	>100	>100	>100
*Pe*	62.82 ± 1.67 ^b^	86.62 ± 0.18 ^a^	>100
*Pf*	>100	95.93 ± 5.47 ^b^	>100
Positive Control	75.55 ± 7.75	49.15 ± 0.96	0.040 ± 0.003

Statistical differences were assessed using ANOVA followed by Tukey’s post hoc test. In columns, different letters next to values indicate significant differences at *p* < 0.05. Positive controls: Epigallocatechin gallate—EGCG (elastase and collagenase); 4-*n*-butylresorcinol (tyrosinase). *Cb*, *Coleus barbatus*; *Cc*, *Coleus cylindraceus*; *Cg*, *Coleus grandidentatus*; *Ch*, *Coleus hadiensis*; *Cm*, *Coleus madagascariensis*; *Pa*, *Plectranthus ambiguus*; *Pe*, *Plectranthus ecklonii*; *Pf*, *Plectranthus fruticosus.*

**Table 8:** Calibration curve parameters.

	Rt/Min	Regression Equation	Correlation Coefficient (R2)
RA	11.5	y = 21301x − 334.07	0.9966

RA—rosmarinic acid.

## Data Availability

The data that support the findings of this study are available from the corresponding author upon request. The data are not publicly available due to privacy and ethical restrictions.

## Abbreviations

The following abbreviations are used in this manuscript:

*C. albicans*	*Candida albicans*
*Cb*	*Coleus barbatus* (Andrews) Benth. ex G. Don (synonym *Plectranthus barbatus*)
*Cc*	*Coleus. cylindraceus* (Hochst. ex Benth.) A.J. Paton (synonym *Plectranthus cylindraceus*)
*Cg*	*Coleus grandidentatus* (Gürke) A.J. Paton (synonym *Plectranthus grandidentatus*)
*Ch*	*Coleus hadiensis* (Forssk.) A.J. Paton (synonym *Plectranthus hadiensis*)
*Cm*	*Coleus madagascariensis* (Pers.) A. Chev. (synonym *Plectranthus madagascariensis*)
ChC	*Clostridium histolyticum* collagenase
DCFH	2,7-dichlorohydrofluorescein
DPPH	2,2-diphenyl-1-picrylhydrazyl
DMEM	Dulbecco’s Modified Eagle Medium
DMSO	Dimethyl sulfoxide
*E. coli*	*Escherichia coli*
EGCG	Epigallocatechin gallate
HBSS	Hanks’ Balanced Salt Solution
HPLC-DAD	high-performance liquid chromatography with a diode array detection
I	elastase inhibition
IC50	Half-Maximal Inhibitory Concentration
*P. acnes*	*Propionibacterium acnes*
*P. aeruginosa*	*Pseudomonas aeruginosa*
*S. aureus*	*Staphylococcus aureus*
*Pa*	*Plectranthus ambiguus*
*Pe*	*Plectranthus ecklonii* Benth.
*Pf*	*Plectranthus fruticosus* L’Hér.
MBC	Minimum Bactericidal Concentration
MDA	malondialdehyde
MIC	Minimum Inhibitory Concentration
mTYR	mushroom tyrosinase
n.t.	Not tested
N.S.	Not specified
na	Not active
NO	nitric oxide
*S. epidermidis*	*Staphylococcus epidermidis*
*S. cerevisiae*	*Saccharomyces cerevisiae*
SANA	N-Succinyl-Ala-Ala-Ala-p-nitroanilide
SRB	sulforhodamine B
TBARS	thiobarbituric Acid Reactive Substances
